# Hypoxic preconditioning rejuvenates mesenchymal stem cells and enhances neuroprotection following intracerebral hemorrhage via the miR-326-mediated autophagy

**DOI:** 10.1186/s13287-021-02480-w

**Published:** 2021-07-22

**Authors:** Jianyang Liu, Jialin He, Lite Ge, Han Xiao, Yan Huang, Liuwang Zeng, Zheng Jiang, Ming Lu, Zhiping Hu

**Affiliations:** 1 Department of Neurology, The Second Xiangya Hospital, Central South University, Changsha, Hunan China; 2 National Health Commission Key Laboratory of Birth Defects Research, Prevention, and Treatment, Hunan Provincial Maternal and Child Health Care Hospital, Changsha, Hunan China; 3 Developmental Biology of Ministry of Education, College of Life Sciences, Hunan Normal University, Changsha, Hunan China; 4 Hunan Provincial Key Laboratory of Neurorestoratology, Second Affiliated Hospital of Hunan Normal University, Changsha, Hunan China

**Keywords:** Mesenchymal stem cells, Intracerebral hemorrhage, Hypoxic preconditioning, Cellular senescence, Autophagy, miRNAs

## Abstract

**Background:**

Intracerebral hemorrhage (ICH) is a major public health concern, and mesenchymal stem cells (MSCs) hold great potential for treating ICH. However, the quantity and quality of MSCs decline in the cerebral niche, limiting the potential efficacy of MSCs. Hypoxic preconditioning is suggested to enhance the survival of MSCs and augment the therapeutic efficacy of MSCs in ICH. MicroRNAs (miRNAs) are known to mediate cellular senescence. However, the precise mechanism by which miRNAs regulate the senescence of hypoxic MSCs remains to be further studied. In the present study, we evaluated whether hypoxic preconditioning enhances the survival and therapeutic effects of olfactory mucosa MSC (OM-MSC) survival and therapeutic effects in ICH and investigated the mechanisms by which miRNA ameliorates hypoxic OM-MSC senescence.

**Methods:**

In the in vivo model, ICH was induced in mice by administration of collagenase IV. At 24 h post-ICH, 5 × 10^5^ normoxia or hypoxia OM-MSCs or saline was administered intracerebrally. The behavioral outcome, neuronal apoptosis, and OM-MSC survival were evaluated. In the in vitro model, OM-MSCs were exposed to hemin. Cellular senescence was examined by evaluating the expressions of P16INK4A, P21, P53, and by β-galactosidase staining. Microarray and bioinformatic analyses were performed to investigate the differences in the miRNA expression profiles between the normoxia and hypoxia OM-MSCs. Autophagy was confirmed using the protein expression levels of LC3, P62, and Beclin-1.

**Results:**

In the in vivo model, transplanted OM-MSCs with hypoxic preconditioning exhibited increased survival and tissue-protective capability. In the in vitro model, hypoxia preconditioning decreased the senescence of OM-MSCs exposed to hemin. Bioinformatic analysis identified that microRNA-326 (miR-326) expression was significantly increased in the hypoxia OM-MSCs compared with that of normoxia OM-MSCs. Upregulation of miR-326 alleviated normoxia OM-MSC senescence, whereas miR-326 downregulation increased hypoxia OM-MSC senescence. Furthermore, we showed that miR-326 alleviated cellular senescence by upregulating autophagy. Mechanistically, miR-326 promoted the autophagy of OM-MSCs via the PI3K signaling pathway by targeting polypyrimidine tract-binding protein 1 (PTBP1).

**Conclusions:**

Our study shows that hypoxic preconditioning delays OM-MSC senescence and augments the therapeutic efficacy of OM-MSCs in ICH by upregulating the miR-326/PTBP1/PI3K-mediated autophagy.

**Supplementary Information:**

The online version contains supplementary material available at 10.1186/s13287-021-02480-w.

## Background

Intracerebral hemorrhage (ICH) causes 15–20% of all strokes and results in higher mortality and disability in the population than ischemic stroke [1]. There is a lack of effective treatment that successfully reduces mortality and disability. Thus, the development of effective treatment options is urgently needed. Cell-based therapy holds the promise of a cure for ICH [2, 3]. Mesenchymal stem cells (MSCs) from various sources, such as the adipose tissue [4, 5], bone marrow [6], umbilical cord tissue [7], and umbilical cord blood [8], have been applied in the treatment of ICH. A previous meta-analysis suggested that stem cell therapy significantly improved behavioral outcomes in an ICH animal model [9]. Olfactory mucosa MSCs (OM-MSCs), located in the nasal lamina propria, are an attractive source of stem cells as they are relatively easy to obtain and ideally suited for autologous transplantation [10]. Previously, we demonstrated that OM-MSCs exert neuroprotective effects in cerebral ischemia/reperfusion injury [11, 12], but no study has explored the neuroprotective effects of OM-MSC therapy in ICH.

Although OM-MSCs hold great potential in regenerative medicine for ICH, recent studies have revealed that transplanted cells survive poorly in the stoke microenvironment [13]. Senescence of stem cells after transplantation is a limiting factor in ICH treatment. Therefore, we sought to develop a method to ameliorate the senescence of MSCs following transplantation with an aim to enhance their therapeutic efficacy in ICH. Hypoxic preconditioning is a suitable approach to enhance stem cell transplantation therapy [14–18]. Stem cells with hypoxic preconditioning present increased viability, prolonged proliferation, and enhanced cell homing [19, 20]. Previously, we demonstrated that hypoxic preconditioning promotes the proliferation of OM-MSCs [21], and the protective effects of OM-MSCs in cerebral ischemia/reperfusion injury [22]. Whether hypoxic preconditioning enhances the therapeutic efficacy of OM-MSCs in ICH remains unclear.

MicroRNAs (miRNAs) are short non-coding RNAs that negatively regulate mRNA expression by binding to the 3′-untranslated region of target mRNAs and playing a role in post-transcriptional silencing [23]. MiRNAs have been proven to play an important role in cellular senescence [24, 25]. Overexpression of miR-1292 in adipose-derived MSCs promoted cellular senescence and restrained osteogenesis [26], overexpression of miR-145a accelerated bone marrow-derived MSCs senescence [27], and miR-378 transfection effectively promoted bone marrow-derived MSC survival [28]. Whether miRNAs play important roles in the senescence of normoxia and hypoxia OM-MSCs remains to be further studied.

Autophagy is a highly conserved catabolic process in which cytoplasmic components are delivered to lysosomes for degradation [29]. Accumulated evidence indicates that autophagy is implicated in MSC senescence [30, 31]. The upregulation of autophagy could delay stem cell aging [32–35]. In addition, some research has suggested that transplanted MSCs present insufficient autophagy in disease microenvironments [36, 37] and that hypoxic preconditioning can improve the autophagy of stem cells [38]. However, it remains uncertain whether adequate autophagy is maintained in transplanted MSCs under ICH conditions.

In the present study, we aimed to investigate the effect of hypoxic preconditioning on the survival and therapeutic efficacy of OM-MSCs in treating ICH. We further clarified the mechanism by which hypoxia preconditioning alleviates the senescence of OM-MSCs. Through microarray and bioinformatic analysis, we investigated the differences in the miRNA expression profiles between normoxia and hypoxia OM-MSCs. We upregulated and downregulated miR-326 to demonstrate its role in hemin-induced OM-MSC senescence. Furthermore, we researched the autophagy in normoxia and hypoxia OM-MSCs and clarified the relations between hypoxic preconditioning, cellular senescence, miR-326, and autophagy. In summary, our study demonstrates a therapeutic method to enhance the efficacy of OM-MSCs in ICH and elaborates the possible mechanism.

## Methods

### Isolation and identification of OM-MSCs

Human OM-MSCs from healthy donors (two males, two females, 20–40 years old) were isolated from the surface interior of the concha nasalis media under otolaryngology endoscopy operation at the Department of Otolaryngologic Surgery, the Second Affiliated Hospital of Hunan Normal University (Changsha, Hunan, China). Informed consent was given to each subject before the operations. The ethics committee of the Hunan Normal University has approved this procedure protocol (Approved No. 2009163009). The olfactory mucosal tissue was isolated and cultured following a published protocol [10]. Passage 3 OM-MSCs were chosen for use in this experiment. After being incubated with 5 mL of monoclonal PE-conjugated antibodies against specific membrane markers (CD105, CD90, CD73, CD44, CD146, CD133, CD34, and CD45; eBioscience, San Diego, CA, USA).

### Hypoxic preconditioning

OM-MSCs were incubated at 37°C under 3% O_2_, 5% CO_2_, and 92% N_2_ for 48 h in a gas-tight humidified chamber (modular incubator chamber; Billups-Rothenberg, Del Mar, CA, USA). Normoxia OM-MSCs were incubated under 21% oxygen and 5% carbon dioxide.

### In vivo experimental design

#### Animals and experimental groups

C57BL/6 mice (male, 14 to 15 weeks old, weighing 25–28 g) were obtained from the animal center of the Hunan Normal University. The mice were housed under controlled environmental conditions (standard lighting conditions, temperature of 20–25 °C and humidity of 40–60%). Experimental protocols were approved by the Animal Care and Use Committee of Hunan Normal University (Approval No.2020-110 164).

#### ICH model in vivo

We used an experimental ICH procedure, described previously [39]. Male mice were anesthetized with 3.5% isoflurane and maintained with 1.0–2.0% isoflurane in 30% oxygen (0.3 L/min) and 70% nitrous oxide (0.7 L/min) mixture. ICH was successfully established by the injection of 0.075 U collagenase IV (C5138; Sigma-Aldrich) dissolved in 1.0 μL of PBS. The position was 0.2 mm anterior, 3.6 mm ventral, and 2.3 mm lateral to the bregma, and the injection rate was 0.1 μl/min. After being left in place for another 10 min, the needle was gently removed to avoid reflux. During the period of modeling and recovery from anesthesia, mice were placed on a heating pad to maintain their body temperature at 37.0°C. Mice in the sham-operated group were subjected to the same procedures without injection of collagenase IV.

#### OM-MSC intracerebral transplantation

For the ICH + MSC-treated group, 5×10^5^ OM-MSCs in 2 μl of saline were stereotactically injected into the ipsilateral lesion area 24 h after ICH at a rate of 0.1 μl/min and left in place for another 10 min. These targets approximated the edge of the bleeding area, with estimated stereotaxic coordinates (0.2 mm anterior, 3.0 mm ventral, and 2.0 mm lateral to the bregma). For the ICH + saline-treated group, 2 μl of saline without OM-MSCs was administered at the same position. In the normoxia MSC group, the mice were injected with non-preconditioned OM-MSCs, and in the hypoxia MSCs group, the mice were injected with hypoxic preconditioned OM-MSCs. The procedure used for intracerebral transplantation was described in previous studies [14, 40]

### Neurobehavioral tests

A standardized battery of behavioral tests was used to quantify neurological function before and 7 days, 14 days, and 28 days after ICH with different treatments. All behavioral tests were performed by two investigators who were blinded to the experimental groups. The 12-point modified neurologic severity score (mNSS) was used to evaluate the sensorimotor integration of forelimbs [41]. The rotarod test was performed using an accelerating rotarod [42]. The speed of rotation (rotation/minute) was increased slowly from 4 to 40 in 5 min. Mice underwent rotarod training for 3 days before ICH establishment.

### Neuronal apoptosis in vivo

#### Tissue preparation

Mice were transcardially perfused with 0.9% ice-cold saline, followed by 4% paraformaldehyde in PBS, pH 7.4. The whole brains were then removed, kept in the same fixative for 4 h at 4°C, and sequentially immersed in 20% and 30% sucrose in 0.1 M phosphate buffer solution until saturated. Coronal sections (10 μm) between +4.7 and −5.2 mm from the bregma were sliced on a cryostat (Leica CM1850) and stored at − 20°C for the subsequent staining.

#### TUNEL assay

Apoptosis in the peri-hematoma area was detected using the terminal deoxynucleotidyl transferase biotin-mediated dUTP Nick-end labeling (TUNEL) staining kit (DeadEnd Fluorometric TUNEL System, Promega, Madison, WI, USA). Subsequently, the neurons were stained with rabbit anti-neuronal specific nuclear protein (NeuN) (1:1000; ABN78; Sigma-Aldrich). Nuclei were stained with 6-diamidino-2-phenylindole (DAPI) (D9542; Sigma-Aldrich). Images were acquired using a fluorescence microscope (Motic, China). For each group, five random fields were counted to calculate the percentage of apoptotic neurons. The results are presented as the number of TUNEL/NeuN double-positive cells divided by the number of DAPI-stained nuclei counted × 100%.

#### Nissl staining

Nissl staining solution was purchased from the Beyotime Biotechnology (Cat. No. C0117; Shanghai, China). Representative images of Nissl-stained brain sections in the cortex regions on days 7, 14, and 28 after ICH were acquired with a high-power light microscope. The number of apoptotic neurons was counted using ImageJ software.

#### Hematoxylin and eosin (HE) staining

HE staining was performed with a staining kit (G1120; Solarbio, Beijing, China). Mouse brain slices were washed three times with PBS and were then incubated with hematoxylin staining solution for 90 s, hydrochloric acid ethanol differentiation solution for 60 s, and eosin staining solution for 60 s.

### In vivo imaging system (IVIS) for detection of GFP-labeled OM-MSCs

Before transplantation, OM-MSCs were transduced with a lentiviral vector encoding the green fluorescent protein (GFP) reporter gene (HonorGene Company; Changsha, China). The transduction efficiency was confirmed using a microscope. Approximately 5×10^5^ GFP-labeled OM-MSCs were implanted in mice via intracerebral administration. On day 14, the mice were sacrificed, whole-brain tissues were resected, and images of the whole brain tissues were acquired. GFP-labeled OM-MSCs were detected with an IVIS Imaging System (IVIS SPECTRUM; Caliper Life Science, Hopkinton, MA, USA) to investigate their engraftment and distribution throughout the brain tissues.

### Simulation of ICH conditions with OM-MSCs in vitro

To simulate conditions of the in vitro ICH model, 1.5 × 10^6^ OM-MSCs were cultured with 100 μM hemin (51280; Sigma-Aldrich). To determine the appropriate duration of hemin treatment, cells were cultured with hemin for 0, 6, 12, 18, and 24 h. The viability of OM-MSCs generally was qualitatively evaluated using a CCK-8 Assay Kit (Dojindo Molecular Technologies) according to the manufacturer’s protocol.

### Transfection and grouping for in vitro experiments

The miR-326 inhibitor and mimics were synthesized by HonorGene Company (Changsha, China). OM-MSCs were seeded into six-well plates 24 h before transfection. When the OM-MSCs were 60% confluent, the miRNA mimics (50 nM) or miRNA inhibitor (100 nM) was transfected with Lipofectamine 3000 according to the manufacturer’s instructions (Invitrogen, Carlsbad, CA, USA). The transfection efficiency was confirmed using RT-qPCR. Twenty-four hours after transfection, OM-MSCs were harvested for the following experiments. The in vitro experimental groups are illustrated in Additional file 1: Table S1.

### 3-Methyladenine and rapamycin treatment

OM-MSCs (1.5 × 10^6^) were treated with 3-methyladenine (3-MA) (5 mmol/L, 6 h, Sigma-Aldrich, USA) or rapamycin (25 nmol/L, 6 h, Cell Signaling Technology, USA) to inhibit or induce autophagy, respectively.

### β-Galactosidase (SA-β-gal) staining

SA-β-gal staining was applied to detect cellular senescence using an SA-β-gal staining kit (C0602; Beyotime, China) according to the manufacturer’s instructions. The percentage of senescent cells was determined after counting cells from ten random fields. Representative fields were imaged with a 40x objective.

### Western blot analysis in vivo and in vitro

Proteins were extracted from OM-MSCs or brain tissue using a total protein extraction kit (P0033; Beyotime Biotechnology). Protein concentration was determined with a BCA Protein Assay Kit (P0010; Beyotime Biotechnology). Protein extracts were separated by SDS-PAGE and transferred to PVDF membranes. The membranes were blocked and incubated with the indicated primary antibodies against P21(1: 1000; 10355-1-AP; Proteintech), P53(1: 2000; 10442-1-AP; Proteintech), P16INK4A (1: 1000; 10883-1-AP; Proteintech), LC3 (1: 1000; 18725-1-AP; Proteintech), P62(1: 2000; 18420-1-AP; Proteintech), Beclin-1(1: 3000; 11306-1-AP; Proteintech), PTBP1 (1:3000; 12582-1-AP; Proteintech), PI3K (1: 1500; ab227204; Abcam), p-PI3K (1: 750; ab182651; Abcam), and actin (mouse, 1:5000; 66009-1-Ig; Proteintech) at 4^°^C overnight. After washing, the membranes were incubated with a horseradish peroxidase-conjugated secondary antibody (1: 5000; anti-mouse or anti-rabbit IgG; SA00001-1; SA00001-2; Proteintech). The membranes were visualized using an ECL detection kit (Bio-Rad, Munich, Germany).

### Total RNA extraction and RNA sequencing analysis

Total RNA was extracted from OM-MSCs using TRIzol (Invitrogen, Carlsbad, CA, USA) according to the manufacturer’s instructions. RNA integrity was analyzed with an Agilent RNA 6000 Chip in an Agilent 2100 Bioanalyser (Agilent Technologies). A microRNA library was then constructed according to the instruction manual, and the final library was qualified, quantified, and sequenced with paired-end reads on the HiSeq X-ten platform (BGI-Shenzhen, China).

Hierarchical cluster heatmaps were generated with pheatmap (v1.0.8) to show the gene expression levels in each sample. Differential expression analysis was performed using DESeq2 (v1.4.5). Differentially expressed genes between hypoxia and normoxia cultured groups were identified as those with |log_2_ (fold change) | > 1; the Q value was obtained after the P value was corrected using the false discovery rate, and a Q value ≤ 0.001 was considered statistically significant. All clean data have been submitted to the SRA database, and the SRA accession is PRJNA624725.

### Quantitative real-time PCR

Total RNA was extracted from OM-MSCs using TRIzol (Invitrogen, Carlsbad, CA, USA) according to the manufacturer’s instructions. MiRNA expression was normalized to that of U6 snRNA. Relative miRNA expression levels were calculated by the 2^-ΔΔCt^ method.

Cell survival in brain tissue samples was determined by the detection of human Alu-sx repeat sequences using qPCR. DNA was extracted from paraffin-embedded tissues according to the manufacturer’s protocols (DP316; Tiangen). The primers for amplification of Alu-sx were F:5′-GGCGCGGTGGCTCACG-3′, R:5′-TTTTTTGAGACGGAGTCTCGCTC-3. Finally, the product was evaluated by electrophoresis in a 2.0% agarose gel containing ethidium bromide.

### Dual-luciferase reporter assays

For luciferase assays, HEK293 T cells were transfected with luciferase reporter plasmids and the miR-326 mimics or controls using Lipofectamine 3000 (Invitrogen, Carlsbad, CA, USA). Luciferase activity was analyzed using a Dual-Luciferase® Reporter Assay System (Promega, Madison, WI, USA).

### Plasmid transfection and construction

The recombinant plasmid p-CMV-2-PTBP1 was constructed, and p-CMV-2-PTBP1 and the empty p-CMV-2 plasmid (HonorGene, Changsha, China) were transfected into OM-MSCs. Transfection was performed with Lipofectamine 3000 (Invitrogen, Carlsbad, CA, USA) according to the manufacturer’s protocol. OM-MSCs were used for experiments 24 h after transfection.

### Statistical analysis

All experiments were performed in at least three replicates. Data are expressed as the mean ± SEM values. Differences between groups were estimated using two-tailed unpaired Student’s t test or ANOVA (two-tailed F test) with the Bonferroni correction for post hoc t tests as appropriate. Statistical analysis was conducted with GraphPad Prism 6 software (La Jolla, CA, USA). Differences with P < 0.05 were considered significant.

## Results

### Identification of normoxic and hypoxic cultured OM-MSCs

**Fig. 1:**
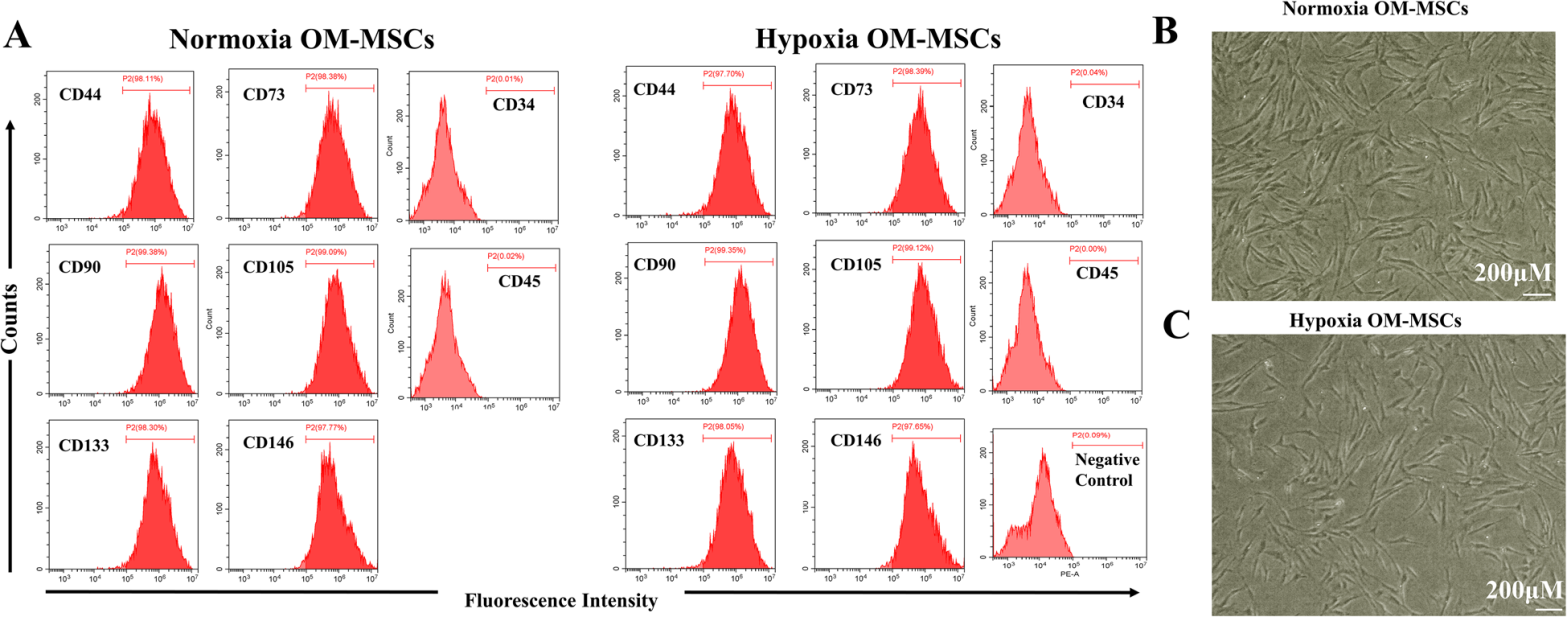
Hypoxia-preconditioned OM-MSCs maintain the stem cell properties. **A** The flow cytometry assesses for the immunophenotypic marker of normoxia and hypoxia OM-MSCs. **B** The morphology of normoxia OM-MSCs in the third passage. **C** The morphology of hypoxia OM-MSCs in the third passage

The expression profiles of surface antigens on hypoxia-preconditioned OM-MSCs were not different from those on normoxic cultured OM-MSCs (Fig. 1A), and the cellular morphology was also unaffected by hypoxic preconditioning (Fig. 1B, C). These results indicate that hypoxia-preconditioned OM-MSCs maintain stem cell properties.

### Hypoxic preconditioning enhanced the neuroprotective effects of OM-MSCs in the ICH model

**Fig. 2:**
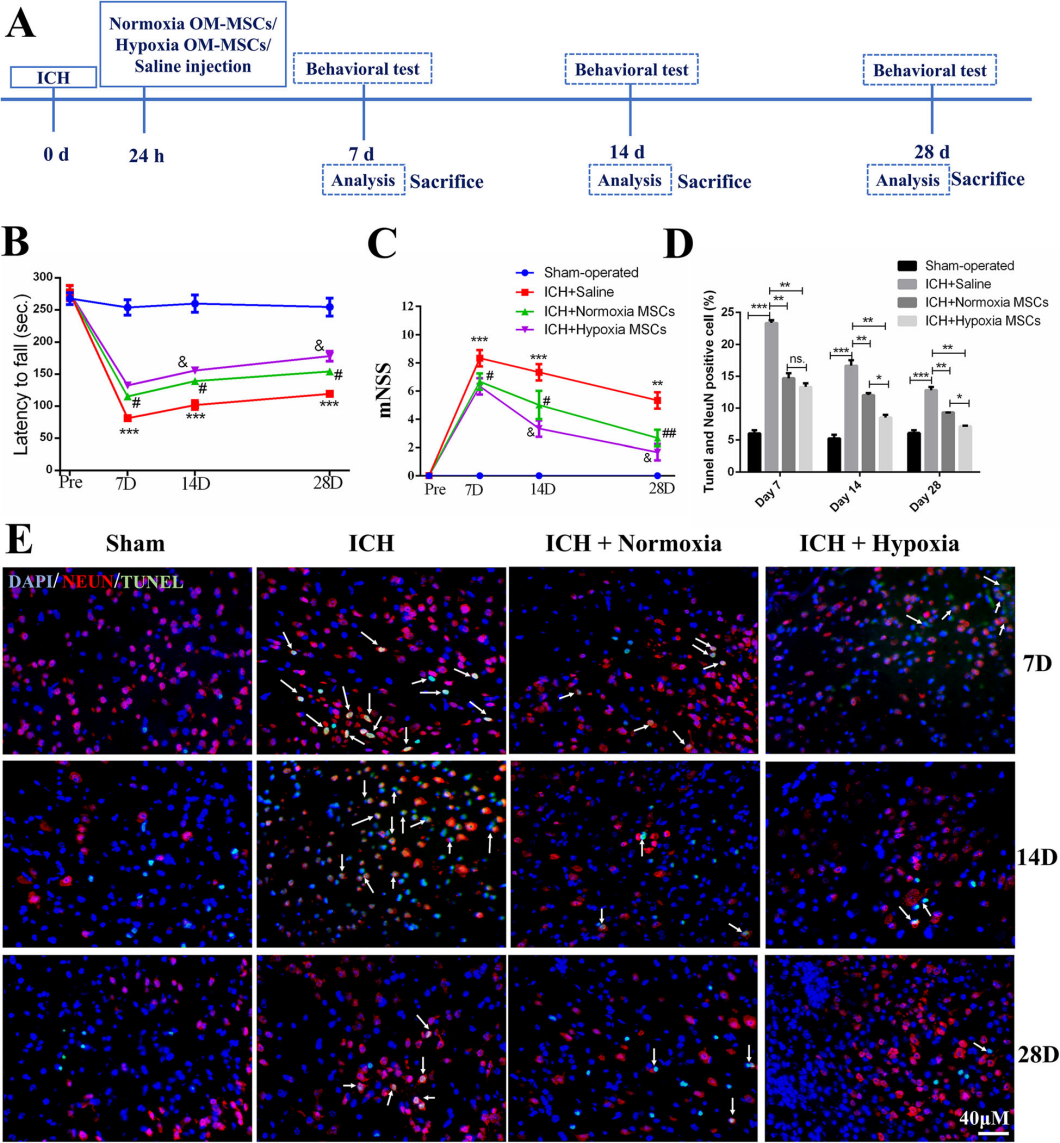
Hypoxic preconditioning augments the neuroprotection of OM-MSCs in ICH animal model. **A** Schematic representation of the experimental design for in vivo experiments. **B** The rotarod test and **C** the modified neurological severity score (mNSS) test were performed before ICH and at 7, 14, and 28 days after ICH. Data are expressed as the mean ± SEM (*n* = 7/group at 7 days; n = 5/group at 14 and 28 days) (^***^P < 0.001, ^∗∗^*P* < 0.01 vs. sham-operated; ^#^*P* < 0.05, ^##^*P* < 0.01 vs. ICH + saline; ^&^*P*< 0.01 vs. ICH + Normoxia MSCs). **D** Quantification of neuronal specific nuclear protein (NeuN) and terminal transferase-mediated dUTP nick end labeling (TUNEL) double-stained cells. Data are expressed as the mean ± SEM (*n* = 4) (^*^P <0.05, ^**^P <0.01, ^***^P <0.001, ns. no significance). **E** Representative images of TUNEL staining in ipsilateral cortex after ICH. The TUNEL and NeuN double-positive cells were indicated with white arrows

OM-MSCs were stereotactically transplanted into the area surrounding the bleed 24 h after ICH. To assess the neuroprotective effects of normoxia and hypoxia OM-MSCs, a series of tests were conducted 7, 14, and 28 days after ICH (Fig. 2A).

The mNSS and rotarod test were used to evaluate the therapeutic effects of OM-MSCs on ICH. Compared with the saline group, the MSC groups, particularly the hypoxia MSC group, showed significant improvements in the rotarod test (Fig. 2B) and the mNSS (Fig. 2C) at 14, and 28 days after ICH. There were no significant differences between the normoxia and hypoxia MSCs group at 7 days after ICH. To investigate neuronal apoptosis after ICH, a TUNEL assay was performed 7, 14, and 28 days after ICH. OM-MSC administration significantly decreased the number of NeuN^+^TUNEL^+^ cells compared with that in the saline group. In addition, hypoxic preconditioning enhanced this protective effect (Fig. 2D, E) at 14, and 28 days after ICH.

**Fig. 3:**
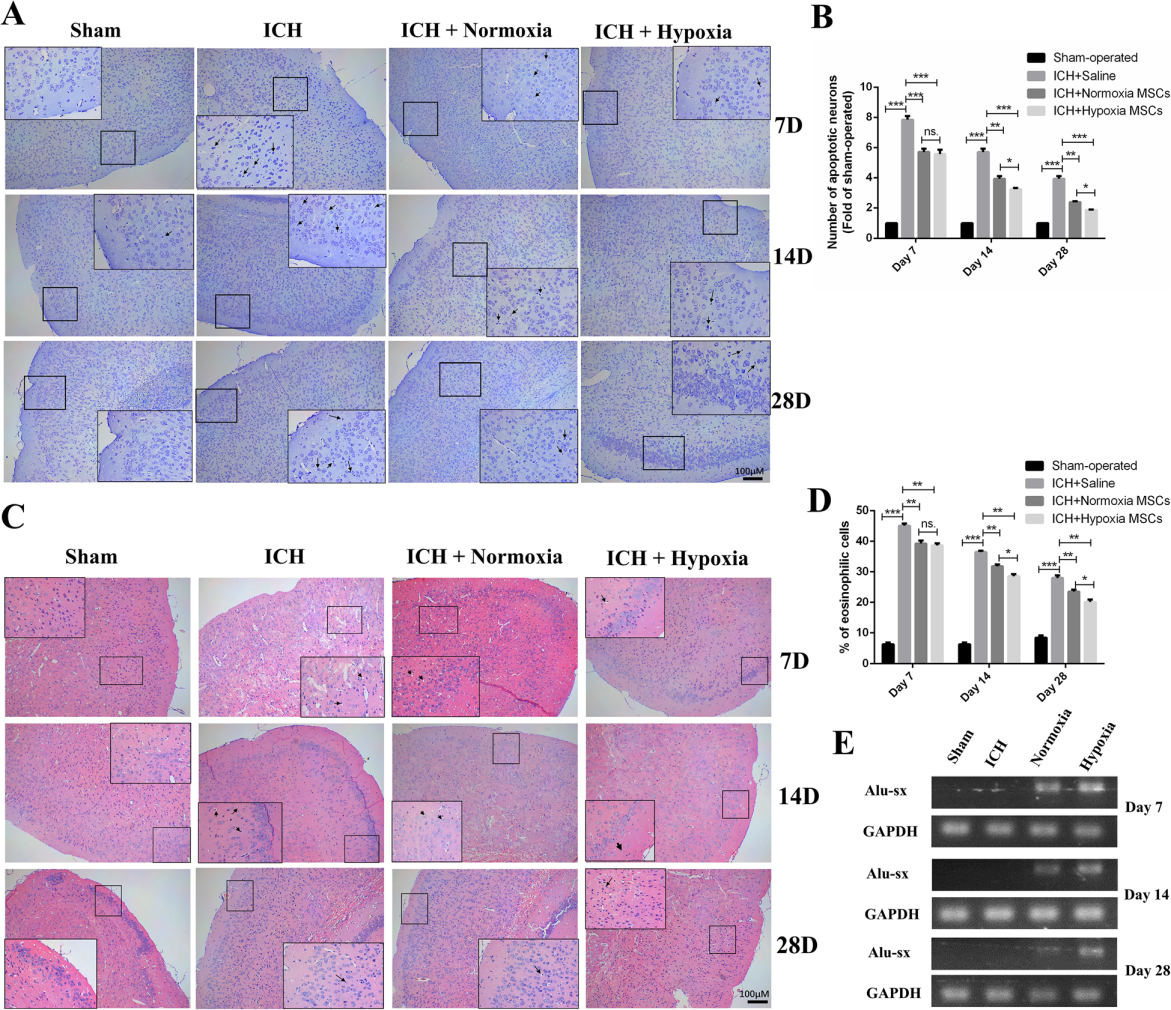
Hypoxic preconditioning promotes the neuroprotection and cellular survival of OM-MSCs in vivo. **A** Representative images of Nissl staining in the cortex regions on days 7, 14, and 28 following ICH. Black arrows indicate apoptotic neurons. **B** Neuronal loss in the cortex and region was significantly ameliorated by OM-MSC treatment, especially the hypoxia OM-MSCs. **C**, **D** HE staining micrographs demonstrate the reduced number of eosinophilic cells in the cortex of ICH mice compared with those in saline group after OM-MSC transplantation. Note that the deep blue nuclei surrounded by white vesicles indicate injured cells (eosinophilic cells, indicated by black arrows), and cells with light blue nuclei are the healthy cells. **E** Representative PCR image of Alu-sx in the brain tissue that received injections of normoxia OM-MSCs, hypoxia OM-MSCs at days 7, 14, and 28 post-ICH. Data are expressed as the mean ± SEM (n = 4) (^*^P <0.05, ^**^P <0.01, ^***^P <0.001, ns. no significance)

In addition to performing a TUNEL assay, we also assessed neuronal apoptosis by Nissl staining and HE staining. Figure 3A shows the images of Nissl staining revealing the neuronal density in the brain with or without treatment with normoxia OM-MSCs or hypoxia OM-MSCs at 7, 14, and 28 days post-ICH. As revealed in Fig. 3B, little neuronal death occurred in the sham-operated group. In the ICH + saline group, extensive neuronal loss was found in the cortex. A decreased number of apoptotic neurons was observed in the brain treated with OM-MSCs, especially hypoxic OM-MSCs on days 14 and days 28 after ICH. Moreover, we applied HE staining to assess the histopathological changes in the cortex. The hypoxia OM-MSC group showed a decrease in the number of eosinophilic cells compared with that in the normoxia OM-MSC group at 14 and 28 days after ICH (Fig. 3C, D).

Generally, both normoxia and hypoxia OM-MSCs reduced the severity of the cerebral injury. Compared to normoxia OM-MSC treatment, hypoxia OM-MSC treatment significantly inhibited neuronal apoptosis at 14 and 28 days post-ICH. The neuroprotective effect in the groups decreased in the order of sham-operated group > hypoxia OM-MSC group > normoxia OM-MSC group > ICH with the saline group.

### OM-MSCs with hypoxic preconditioning showed improved survival after transplantation

Considering the significantly improved therapeutic efficiency of hypoxic OM-MSCs compared with normoxic OM-MSCs at 14 and 28 days post-ICH (p < 0.05), we hypothesized that hypoxic preconditioning may increase the survival of OM-MSCs post-transplantation. To explore whether the effect of hypoxic OM-MSCs on ICH is related to cellular senescence, we investigated the survival of OM-MSCS post-transplantation. The survival of transplanted OM-MSCs was evaluated by the detection of the human repeat sequence Alu-sx in the brain [43]. In the in vivo model, OM-MSCs with hypoxic preconditioning exhibited greater survival 14 and 28 days after ICH (Fig. 3E). GFP-labeled OM-MSCs were intracerebrally administered to mice (Figure S1A), and observation using the IVIS was performed until 14 days after administration. Ex vivo imaging of the excised organs revealed an accumulation of fluorescence in the brain tissues. No fluorescence was noted in the other organs, such as the heart, lung, liver, spleen, and kidney (Figure S1B). Comparison of the relative fluorescence units in the excised tissue of the brains administered normoxia OM-MSCs and hypoxia OM-MSCs revealed that the fluorescence intensity in the hypoxic OM-MSC group was stronger than that in the normoxic OM-MSC group (Figure S1C).

### Hypoxic preconditioning alleviates cellular senescence of hemin-treated OM-MSCs

**Fig. 4:**
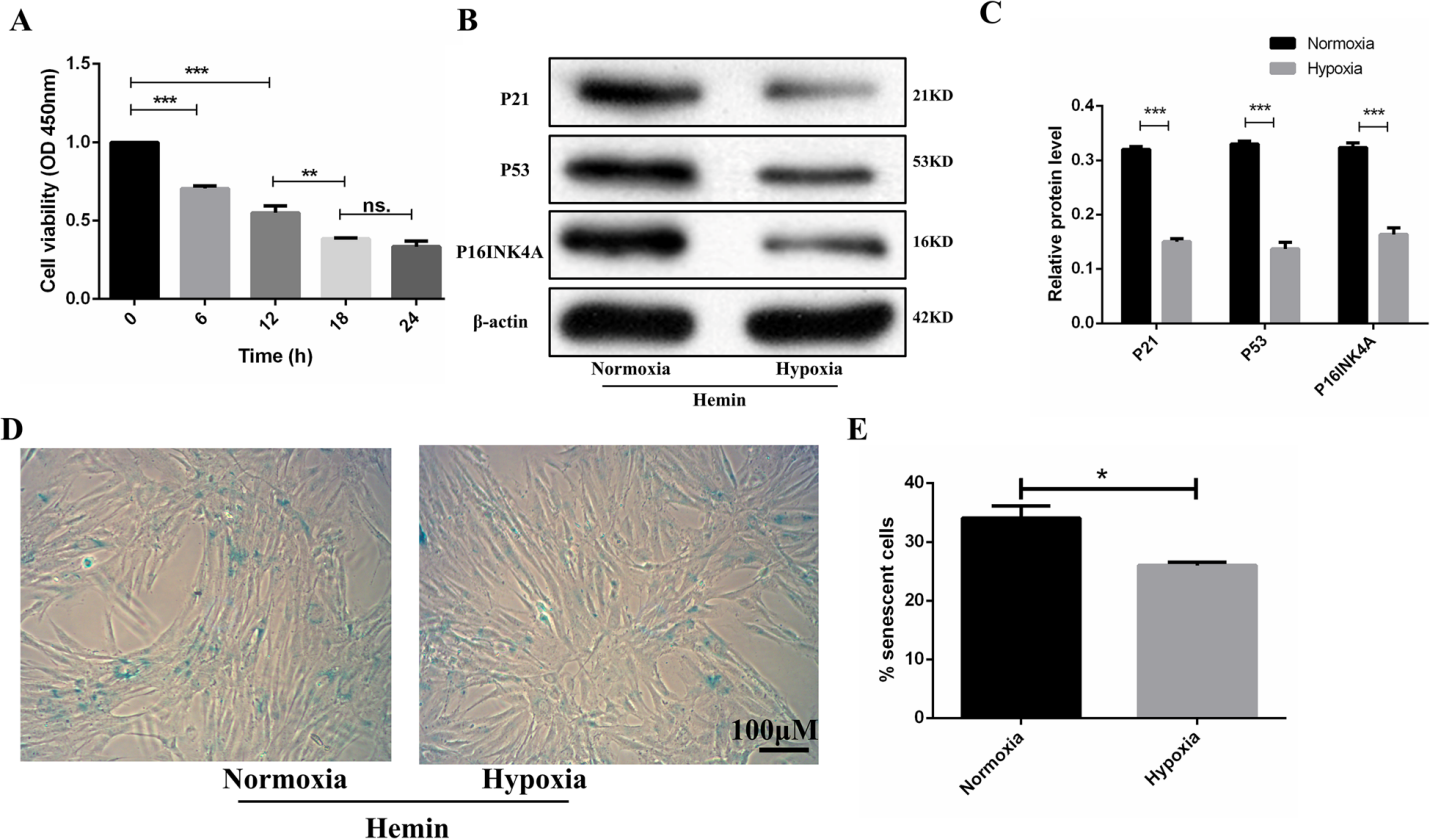
Hypoxic preconditioning alleviates the cellular senescence of OM-MSCs in vitro model. **A** Viability of OM-MSCs treated with Hemin for different lengths of time. **B**, **C** Western blotting for p21, P53, and p16INK4A in normoxia and hypoxia OM-MSCs treated with hemin for 18 h. **D** Representative images of SA-β-gal activity staining. **E** Number of SA-β-gal-positive cells. Data are expressed as the mean ± SEM (*n* = 3) (^*^P <0.05, ^**^P <0.01, ^***^P <0.001, ns. no significance)

To investigate whether hypoxic preconditioning can decrease the senescence of OM-MSCs in an in vitro model, we detected the level of senescence of OM-MSCs treated with hemin. First, we examined the effect of hemin on OM-MSC survival using a CCK-8 assay. After treatment with 100 μM Hemin, cell viability decreased in a time-dependent manner, reaching a minimum at 18 h (Fig. 4A). Thus, we chose the 18 h treatment time for the following study. In the in vitro model, hypoxic preconditioning decreased the protein expression levels of p16INK4A, p21, and p53 (Fig. 4B, C) and the level of SA-β-gal activity (Fig. 4D, E) in OM-MSCs treated with hemin. Collectively, after incubation with hemin, the level of cellular senescence in hypoxia preconditioned OM-MSCs was lower than that in normoxia OM-MSCs.

### miR-326 attenuated the senescence of OM-MSCs in the in vitro model

**Fig. 5:**
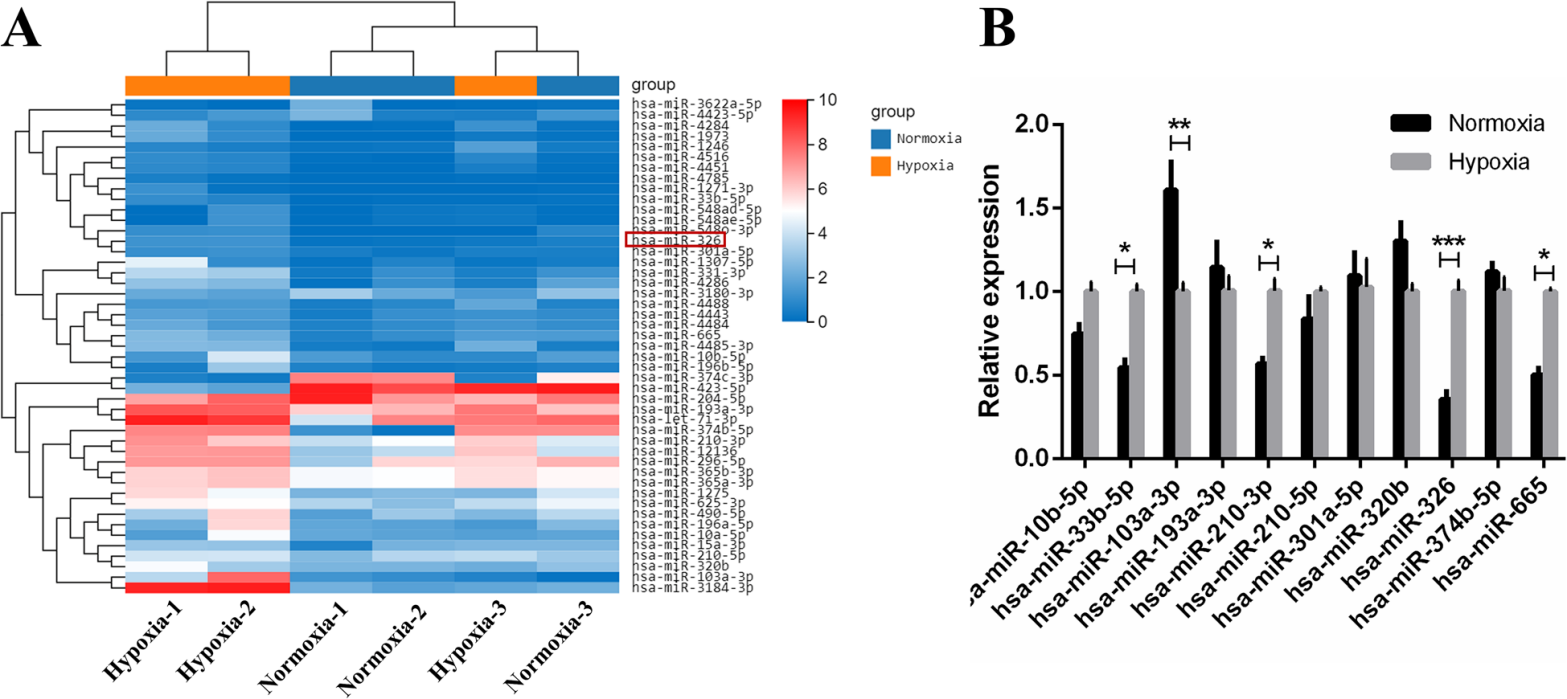
The expression profiles of DE-miRNAs in OM-MSCs under hypoxic and normoxic conditions. **A** Hierarchical clustering was performed on differentially expressed microRNAs between hypoxic OM-MSCs and normoxia OM-MSCs. The results were standardized by log(value+1). **B** Expression of selected OM-MSCs miRNAs was verified by quantitative real-time PCR. Data are expressed as the mean ± SEM (*n* = 3) (^*^P <0.05, ^**^P <0.01, ^***^P <0.001, ns. no significance)

To elucidate why hypoxic preconditioning alleviated the senescence of OM-MSCs in the in vitro model, we compared the miRNA profiles of normoxia OM-MSCs and hypoxia OM-MSCs using microarray and bioinformatic analysis. Differentially expressed genes between the hypoxia and normoxia cultured group were identified as those with |log_2_ (fold change) | > 1.0. Among differentially expressed miRNAs, 41 miRNAs were upregulated, and 6 miRNAs were downregulated with an expression fold change of greater than 2.0 in hypoxia OM-MSCs (Fig. 5A). The expression of selected differentially expressed miRNAs was verified by qRT-PCR, showing that miR-33b-5p, miR-103a-3p, and miR-665 were upregulated in hypoxia OM-MSCs compared to normoxic OM-MSCs (Fig. 5B). The expression of miR-326 was obviously upregulated after hypoxic preconditioning (*p*<0.001). Previous researches have not shown that miR-326 is involved in the regulation of cellular senescence. However, a previous study demonstrated that miR-326 overexpressing endothelial progenitor cells could enhance therapeutic angiogenesis in myocardial infarction [44]. MiR-326 overexpression has been found to inhibit apoptosis and promote the proliferation of neurons in cerebral ischemia-reperfusion injury [45], Parkinson’s disease [46, 47], and Alzheimer’s disease [48]. Therefore, we selected this miRNA for further study.

**Fig. 6:**
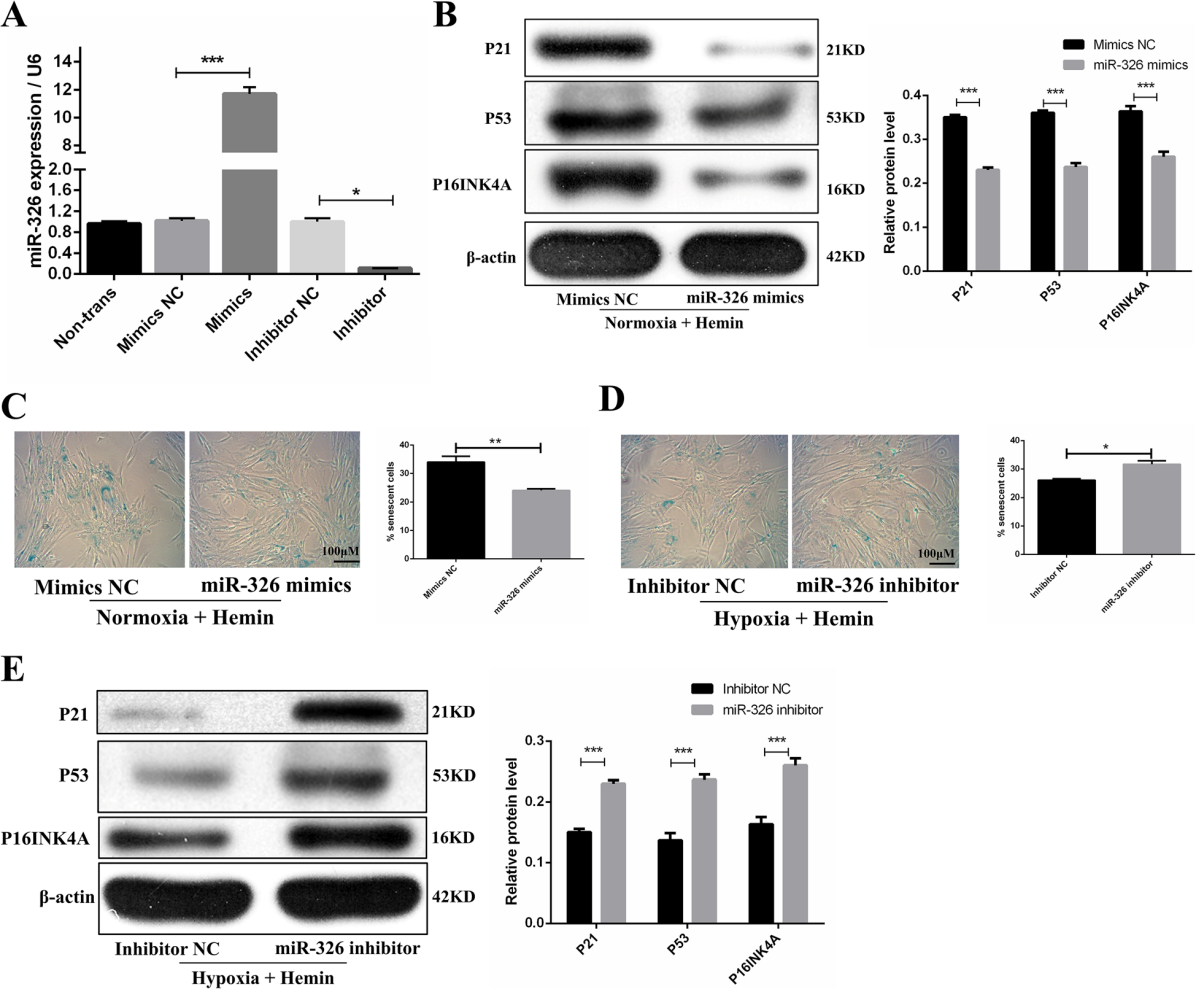
Hypoxic preconditioning attenuates cellular senescence of OM-MSCs by upregulating miR-326 in vitro model. **A** Expression of miR-326 in OM-MSCs after transfection of mimics and inhibitors. **B** Under the hemin treatment, western blotting and quantitative analysis of the expression levels of p21, P53, and p16INK4A in normoxia OM-MSCs transfected with miRNA mimics control or miR-326 mimics. **C** Representative images of SA-β-gal staining and quantitative analysis of SA-β-gal-positive normoxia OM-MSCs transfected with miRNA mimics control or miR-326 mimics. **D** Representative images of SA-β-gal staining and quantitative analysis of SA-β-gal-positive hypoxia OM-MSCs transfected with miRNA inhibitor control or miR-326 inhibitor. **E** Western blotting and quantitative analysis of the expression levels of p21, P53, and p16INK4A in hypoxia OM-MSCs transfected with miRNA inhibitor control or miR-326 inhibitor. Data are expressed as the mean ± SEM (*n* = 3) (^*^P <0.05, ^**^P <0.01, ^***^P <0.001)

To investigate the effects of miR-326 on the cellular senescence of normoxia and hypoxia OM-MSCs, we compared the cellular senescence of miR-326 mimics, inhibitors, or negative control-transfected OM-MSCs under hemin treatment. The transfection efficiency was proven by qRT-PCR, and the results suggested that transfection of the miR-326 mimics elevated the expression of miR-326 more than 10-fold and transfection of inhibitors of miR-326 reduced the expression of miR-326 by approximately 70% compared with that in cells transfected with the corresponding negative control (Fig. 6A). We then showed that the miR-326 mimics decreased the protein expression levels of p16INK4A, p21, and p53 (Fig. 6B) and the level of SA-β-gal activity (Fig. 6C) in normoxic OM-MSCs with hemin treatment, whereas miR-326 inhibitors presented the opposite effects in hypoxia OM-MSCs in the condition of hemin treatment (Fig. 6D, E). These observations suggested that hypoxic preconditioning alleviated OM-MSC senescence in the in vitro model, an effect that was partially ascribed to upregulation of miR-326.

### miR-326 reduced cellular senescence of hypoxia-preconditioned OM-MSCs by regulating autophagy

**Fig. 7:**
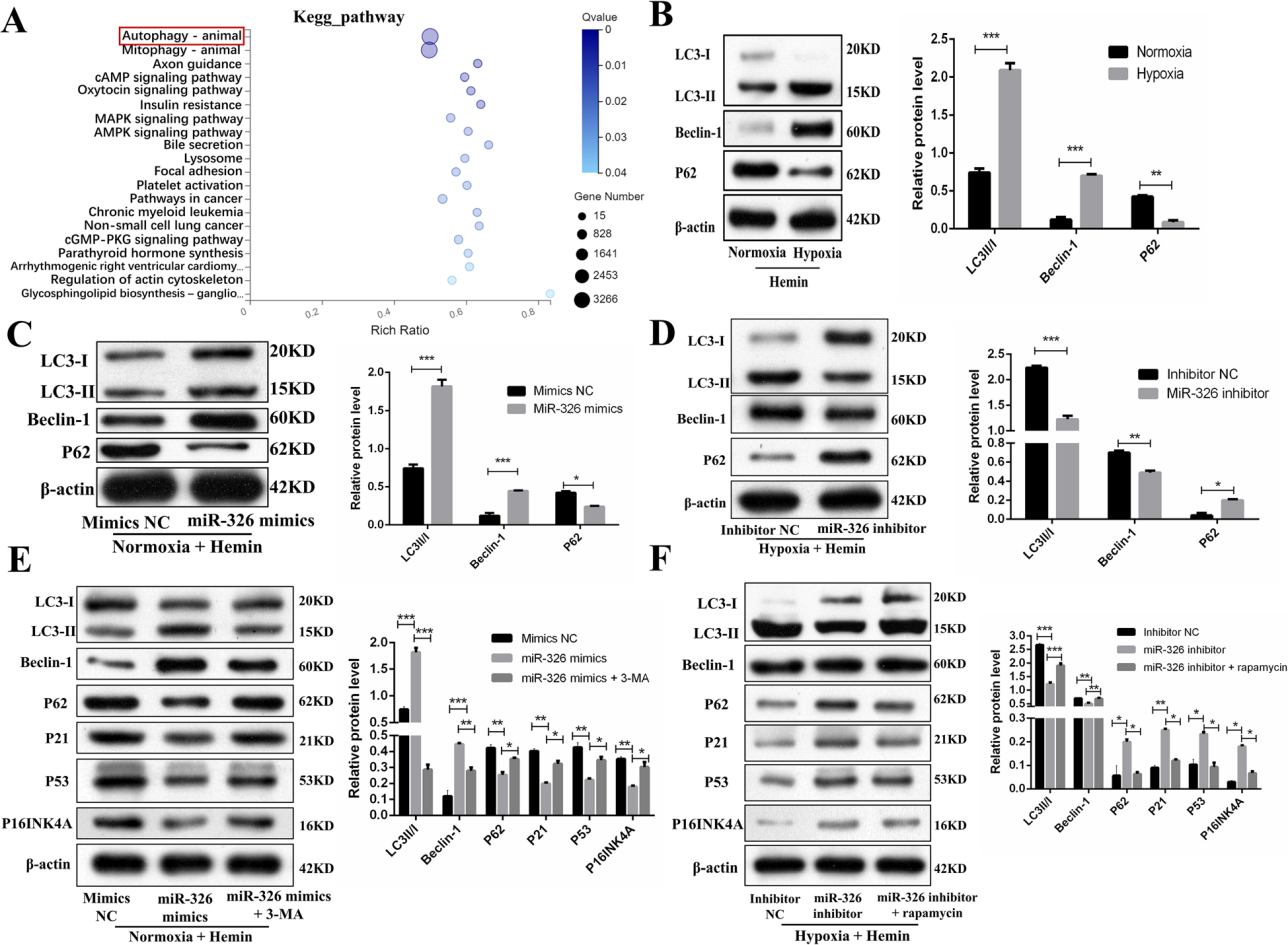
miR-326 attenuates cellular senescence of hypoxia OM-MSCs by promoting autophagy in vitro model. **A** Kyoto Encyclopedia of Genes and Genomes (KEGG) pathway analyses of miR-326. **B** Western blotting and quantitative analysis of the expression levels of LC3II/I, Bcelin-1, and P62 in Hemin-exposed normoxia and hypoxia OM-MSCs. **C** Western blotting and quantitative analysis of the expression levels of LC3II/I, Bcelin-1, and P62 in normoxia OM-MSCs transfected with miRNA mimics control or miR-326 mimics. **D** Western blotting and quantitative analysis of the expression levels of LC3II/I, Bcelin-1, and P62 in hypoxia OM-MSCs transfected with miRNA inhibitor control or miR-326 inhibitor. **E** Western blotting analysis of LC3II/I, Bcelin-1, P62, p21, P53, and p16INK4A protein expression in normoxia MSCs transfected with miRNA mimics control, or miR-326 mimics, or miR-326 mimics +3MA. **F** Western blotting analysis of LC3II/I, Bcelin-1, P62, p21, P53, and p16INK4A protein expression in hypoxia MSCs transfected with miRNA inhibitor control, or miR-326 inhibitor, or miR-326 inhibitor + rapamycin. Data are expressed as the mean ± SEM (*n* = 3) (^*^P <0.05, ^**^P <0.01, ^***^P <0.001)

Previous studies have shown that autophagy plays a critical role in the senescence of MSCs and that reducing autophagy diminishes the tolerance of stem cells under various stresses [35, 38, 49]. Pre-activation of autophagy with rapamycin was shown to promote the survival of MSCs in the infarcted myocardium [33]. Moreover, the previous studies [46, 50] and KEGG pathway (Fig. 7A) suggested that miR-326 participates in the regulation of autophagy. Thus, we explored whether miR-326 reduces the cellular senescence of OM-MSCs by upregulating autophagy. First, we examined the level of autophagy in normoxia MSCs and hypoxia MSCs treated by hemin. Western blotting analysis showed that compared with normoxia MSCs, hypoxic MSCs exhibited downregulated expression of p62 and upregulated expression of LC3II/I and Beclin-1 (Fig. 7B). Second, we treated normoxia-MSCs with the miR-326 mimics and discovered that the miR-326 mimics promoted autophagy in normoxia MSCs (Fig. 7C). However, the alleviation of senescence in normoxia MSCs was partially reversed by treatment with 3-MA (an autophagy inhibitor), as evidenced by the increased expression of p16INK4A, p53, and p21 (Fig. 7E). Third, the miR-326 inhibitor decreased autophagy of hypoxia MSCs (Fig. 7D). Notably, the increased cellular senescence in hypoxia-MSCs induced by the miR-326 inhibitor was partially reversed by treatment with rapamycin (an autophagy inducer) (Fig. 7F). Collectively, these results suggested that miR-326 regulates the senescence of OM-MSCs by upregulating autophagy. Based on the increased autophagy in hypoxia OM-MSCs compared with normoxia OM-MSCs, we suggested that hypoxic preconditioning ameliorating OM-MSCs senescence partially attributes to the miR-326-mediated autophagy.

### miR-326 regulated autophagy via the PTBP1/PI3K signaling pathway

**Fig. 8:**
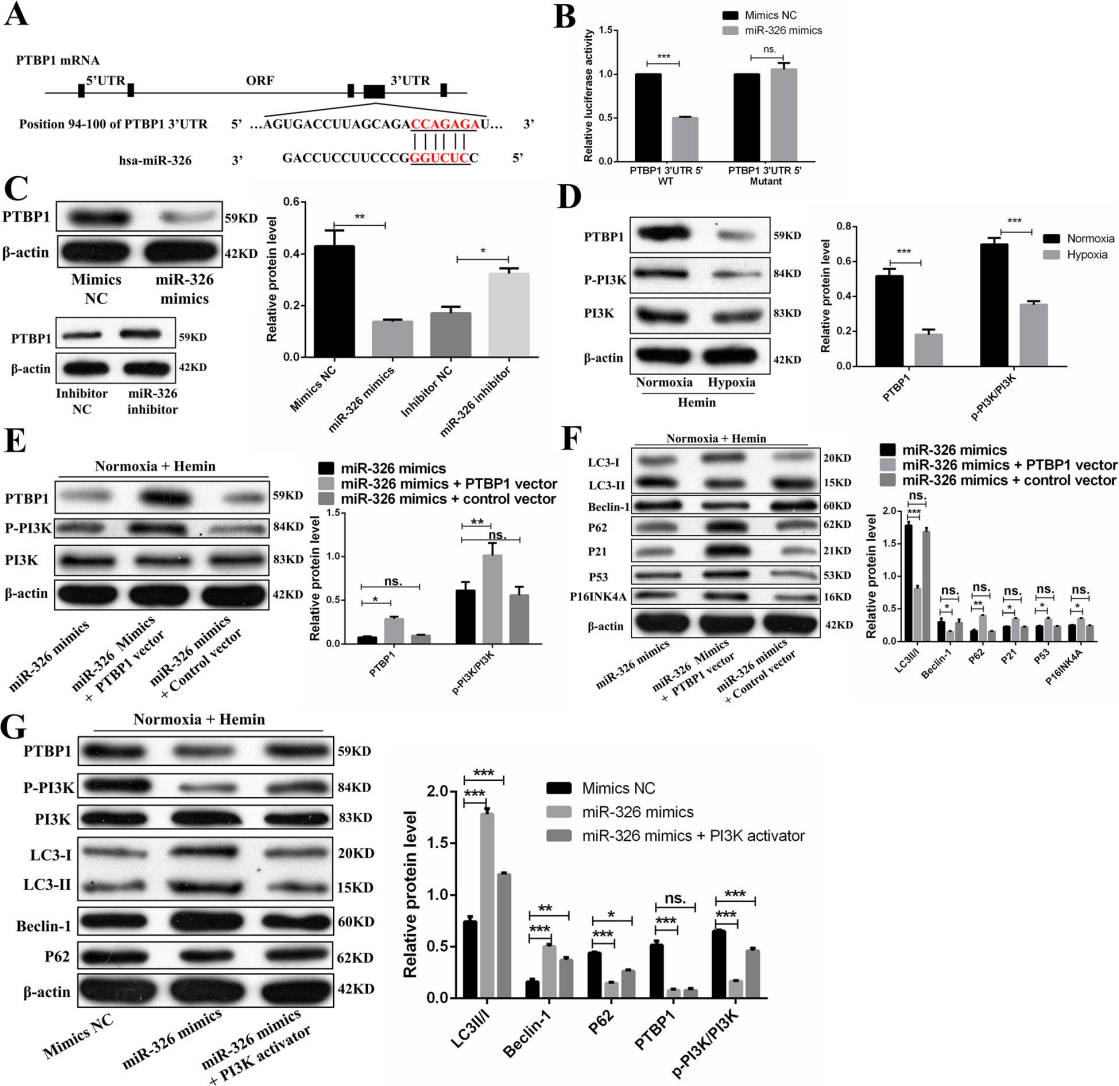
miR-326 promotes autophagy via the PTBP1/PI3K signaling pathway. **A** The potential binding sites for miR-326 on the 3′UTR of PTBP1. **B** 293T cells were co-transfected with miR-326 mimic or miRNA control and with a luciferase reporter vector containing WT or mutant 3′UTR of PTBP1. **C** The expression level of PTBP1 negatively correlated with the level of miR-326 in OM-MSCs. **D** Compared with those in Hemin-exposed normoxia OM-MSCs, the expression levels of p-PI3K/PI3K and PTBP1 were robustly decreased in Hemin-exposed hypoxia OM-MSCs. **E** Overexpressed PTBP1 increased the expression of PTBP1 and p-PI3K/PI3K. **F** Under miR-326 mimics treatment, overexpressed PTBP1 increased the expression of p21, P53, p16INK4A, and P62 protein and decreased the expression of LC3II/I and Bcelin-1 protein. **G** Treatment with PI3K activator partially reversed the downregulation of p-PI3K/PI3K and P63 and the upregulation of LC3II/I and Bcelin-1 induced by the miR-326 mimics. Data are expressed as the mean ± SEM (*n* = 3) (^*^P <0.05, ^**^P <0.01, ^***^P <0.001, ns. no significance)

Previous studies have shown that the PI3K signaling pathway regulates autophagy [51, 52]. Thus, we aimed to investigate whether miR-326 regulates autophagy via the PI3K pathway. We used TargetScan and mircroRNA.org to predict the target genes of miR-326 and found a potential binding site in the 3′UTR of PTBP1. It has also been reported that PTBP1 can affect PI3K activation to downregulate autophagy [53]. The dual-luciferase reporter assay confirmed that the miR-326 mimics resulted in significant downregulation of PTBP1 wild-type (WT) luciferase reporter activity. Upon comparing PTBP1-WT alone and PTBP1 mutant with the miR-326 mimics co-transfection groups, no significant difference was noted (Fig. 8A, B).

First, we demonstrated that the expression level of PTBP1 negatively correlated with the level of miR-326 in OM-MSCs by western blotting assay (Fig. 8C). Second, we estimated the protein levels of PTBP1 and p-PI3K/PI3K in normoxia MSCs and hypoxia MSCs under hemin treatment. Compared with those in normoxia MSCs, the protein levels of PTBP1 and p-PI3K/PI3K in hypoxia MSCs were greatly decreased (Fig. 8D). To further verify the role of PTBP1 in miR-326-mediated OM-MSC senescence, we treated MSCs with the miR-326 mimic and then transfected them with the PTBP1 expression vector in normoxia MSCs. As shown in Fig. 8E, F, PTBP1 overexpression decreased autophagy in miR-326 mimics-treated normoxia MSCs. Furthermore, PTBP1 overexpression increased normoxic MSC senescence, which was decreased by miR-326 mimics transfection (Fig. 8F). MiR-326 mimics treatment downregulated the levels of p-PI3K/PI3K and upregulated autophagy (Fig. 8G). However, treatment with 740 Y-P (PI3K agonist) partially reversed the downregulation of PI3K phosphorylation and the upregulation of autophagy induced by the miR-326 mimics (Fig. 8G). In summary, miR-326 promoted autophagy in hemin-exposed OM-MSCs by targeting the PTBP1/PI3K signaling pathway.

### Discussion

MSCs have emerged as a promising therapeutic tool in ICH. Although naïve MSCs are still the most common approach used to treat ICH, researches are being conducted to discover several methods to culture more functional MSCs, including pharmacologic pre-conditioning [54], alternative cell delivery approaches [55–57], genetic modification [58, 59], physical methods [60], and modification of culture conditions [14, 17]. Hypoxic preconditioning is a modification of culture conditions that have been shown to improve the therapeutic efficacy of stem cells in a model of atherosclerotic renal artery stenosis [61], spinal cord injury [62], ischemic stroke [18, 63, 64], acute lung injury [65], myocardial infarction [15], chronic liver injury [66], urethral injury [67], and ICH [14, 17]. The oxygen concentrations evaluated in most of these studies include 0.1–0.3% O_2_, 1% O_2_, 3% O_2_, and 5% O_2_, and the treatment durations range from 24 to 48 h. In a general setting, MSCs are cultured under normoxia niche (~21%) [68]. However, compared to normal brain tissues (~5%), the ICH microenvironment is known to be hypoxic [69]. Thus, hypoxic preconditioning can emulate physiological conditions, may activate the adaptive processes of MSCs under physiological conditions, and can profoundly improve the MSC microenvironment to favor the success of MSCs engraftment. Previous studies have demonstrated that hypoxic preconditioning of bone marrow-derived mesenchymal stem cells [17] and hypoxic preconditioning of neural stem cells [14] enhanced the neuro-regenerative effects and promoted neurological functional recovery after ICH. The possible mechanism involves the paracrine action, increased neurogenesis, and increased cell survival in vivo. The present study investigated the hypothesis that hypoxic preconditioning can improve the therapeutic effects of OM-MSCs in mice subjected to ICH by upregulating the survival of OM-MSCs. First, we evaluated the neurobehavioral function and neuronal apoptosis, and the results showed that compared with normoxia OM-MSC transplantation, hypoxic preconditioning improved neurologic deficit symptoms, and alleviated neuronal apoptosis. Second, we also found that there was no significant difference in the therapeutic effects between normoxia cultured and hypoxia-preconditioned OM-MSCs on day 7 after ICH. However, on day 14 and day 28 after ICH, neurobehavioral deficit and neuronal apoptosis were significantly ameliorated in the hypoxic OM-MSC group compared with the normoxic OM-MSC group. Accordingly, we hypothesized that the improved therapeutic effects of hypoxic OM-MSCs may involve the increased survival of OM-MSCs in vivo. Third, the survival of stem cells is regulated through a variety of processes, including proliferation and differentiation, apoptosis, and cellular senescence [70]. In an in vitro model of ICH, we demonstrated that hypoxic preconditioning alleviated the cellular senescence of OM-MSCs. However, the precise mechanism by which hypoxic preconditioning regulates cellular senescence of OM-MSCs remains unclear.

MiRNAs, endogenous small noncoding RNA molecules, have been reported to play crucial roles in mediating the cellular senescence of stem cells [24, 25, 71], examples include microRNA-206 [15], let-7, miRNA-23a, miRNA-26a, miRNA-30a [72], microRNA-34a-3p [73], miRNA-145a [27], miRNA-1292 [26], miRNA-15a/15b [74], miRNA-155-5p [43], and miRNA-199a-5p [49]. Using microarray and bioinformatic analysis, we compared miRNA expression levels in OM-MSCs cultured in normoxia and hypoxia. We found that the expression of miR-326 in hypoxia OM-MSCs was significantly upregulated. Although no studies have evaluated the role of miR-326 in the cellular senescence of MSCs, studies have reported that miR-326 can inhibit the apoptosis of dopaminergic neurons in Parkinson’s disease mice [47] and increase neuron cell ability in Alzheimer’s disease mice [48]. Overexpression of miR-326 was found to improve the therapeutic effect of human umbilical cord mesenchymal stem cell-derived exosomes in mice with inflammatory bowel disease [75]. MiR-326-5p also significantly enhanced the angiogenic capacity of endothelial progenitor cells in ischemic heart diseases [44]. The results of our experiments demonstrate that miR-326 plays a major role in the reduced cellular senescence of hypoxia OM-MSCs. However, the precise mechanism of miR-326 in regulating hypoxia OM-MSCs senescence remains to be further studied. Previous studies suggested that miR-326 activates autophagy of dopaminergic neurons [46] and promotes autophagic activity in fibroblasts by targeting PTBP1 [50]. Thus, we evaluated the role of autophagy in the cellular senescence of OM-MSCs in the subsequent experiments.

**Fig. 9:**
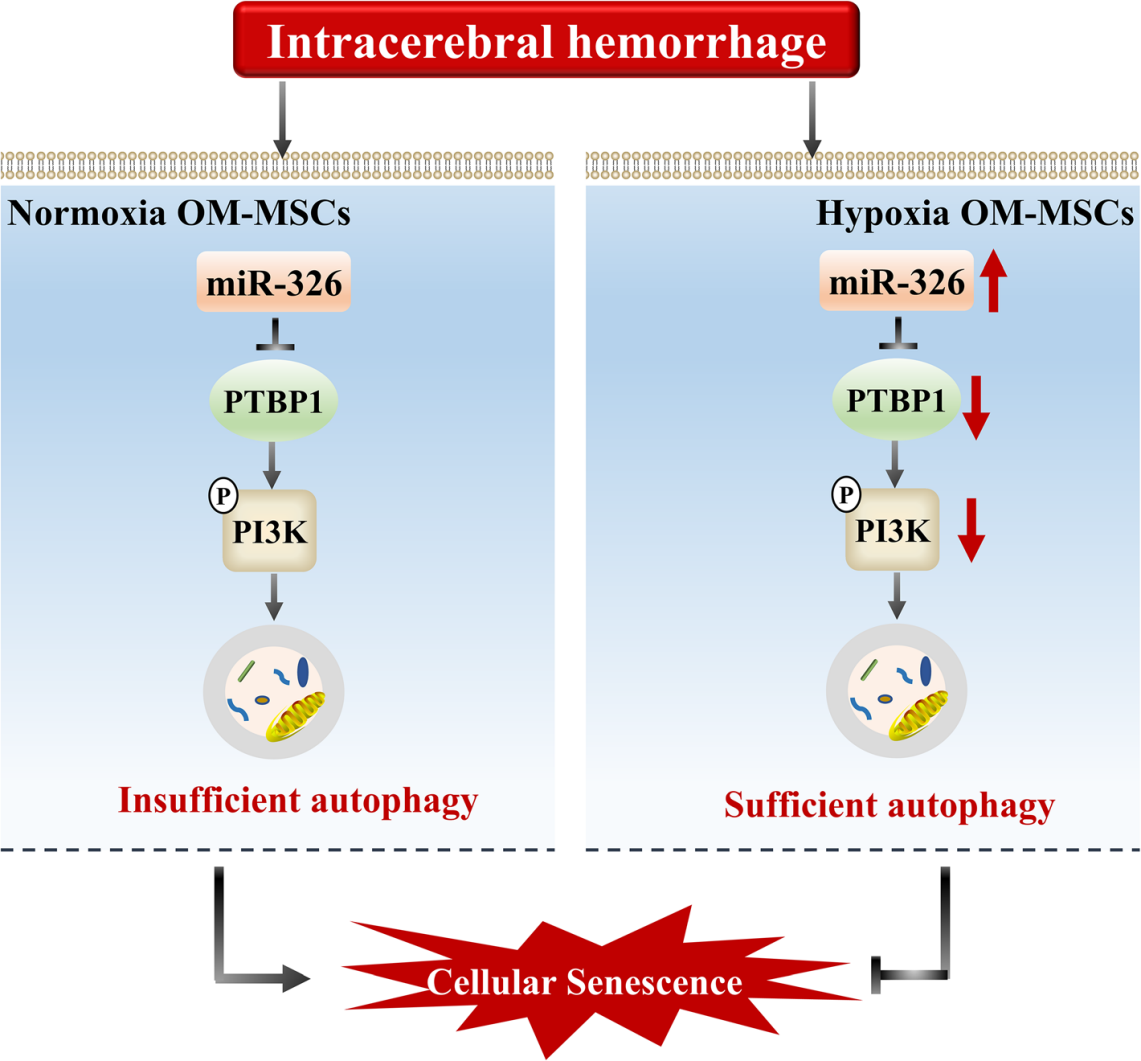
Proposed mechanism for how hypoxic preconditioning rejuvenates OM-MSCs against ICH. In the microenvironment of ICH, the basal autophagy in normoxia OM-MSCs is insufficient, which accelerates the cellular senescence of transplanted OM-MSCs. Compared with normoxia OM-MSCs, hypoxic preconditioning upregulates the expression of miR-326 in OM-MSCs. miR-326 could promote autophagy by targeting PTBP1/PI3K signaling pathway. The sufficient autophagy can maintain OM-MSC functional survival. As a result, hypoxic preconditioning delays OM-MSC senescence and augments the therapeutic efficacy of OM-MSCs in ICH.

Autophagy is a stress response essential for MSC homeostasis. Previous studies reviewed the relations between cellular senescence and autophagy [30, 31] and suggested that autophagy plays a dual role in the regulation of MSC senescence. Upregulation of autophagy can decrease the apoptosis of aged MSCs in the condition of myocardial infarction [34, 35], restore the biological properties of aged MSCs [76], rejuvenate MSCs derived from patients with idiopathic pulmonary fibrosis [49], and reduce the senescence of patellar tendon stem/progenitor cells [77]. Compared with young MSCs, aged MSCs have significantly reduced autophagic activity. The autophagic activity of MSCs is also insufficient under pathological conditions [36, 78]. Autophagy, a fundamental cellular process, helps cells remove degenerated or senescent organelles and accommodate the changing environment. However, the cytoprotective effect of autophagy may be decisive when MSCs are engrafted into areas with a severe oxidative environment [79]. Therefore, there is a need for pre-conditioning strategies to enhance the autophagy of MSCs under pathological conditions. Hypoxic preconditioning was shown to promote the survival of engrafted endothelial progenitor cells in limb ischemia through the induction of autophagy [38]. Hypoxic preconditioning was also found to improve the functional survival and therapeutic efficiency of engrafted MSCs in a model of myocardial infarction, at least in part through autophagy upregulation [80]. The possible mechanisms may involve the leptin [81] and hypoxia-inducible factor 1α [32], which may be important factors in the regulation of hypoxic preconditioning-induced autophagy. Based on the relations among hypoxic preconditioning, cellular senescence, and autophagy, we speculated that the increased cell survival of hypoxia-preconditioned OM-MSCs in the ICH model is related to the induction of autophagy. First, we found that the autophagy level in hemin-treated OM-MSCs was insufficient. Under the ICH environment, the autophagic activity of hypoxia OM-MSCs was increased compared with that of normoxia OM-MSCs. Second, the upregulation of autophagy mediated by miR-326 delayed the senescence of hypoxia-preconditioned OM-MSCs in the ICH model. Third, miR-326 upregulated the autophagy by targeting the PTBP1/PI3K pathway (Fig. 9). These results may indicate one of the important mechanisms for the improved effects of hypoxia-preconditioned OM-MSCs in the ICH model.

There were several limitations in this study. First, a single transplantation time point, a single administration route, and a fixed cell dosing regimen were used. Second, only the mNSS and rotarod test were applied, and these tests might not be optimal for the long-term assessment of neurobehavioral function after ICH. Third, it remains unclear whether differentially expressed miRNAs other than miR-326 play a pivotal role in hypoxic OM-MSCs.

## Conclusions

This study provided a strategy to enhance the neuroprotective effects of OM-MSCs in a model of ICH and demonstrated that hypoxic preconditioning enhances OM-MSC survival under the ICH microenvironment through the induction of miR-326/PTBP1/PI3K-mediated autophagy.

## Supplementary information

**Additional file 1: Table S1.** Groups used for the in-vitro experiments.

**Additional file 2: Figure S1.** Detection of OM-MSCs with an in vivo imaging system (IVIS). **(A)**. The injection site of collagenase IV and OM-MSCs. **(B)**. In the excised organs at 14 day after injection, strong fluorescent signals were observed in the brain tissues. No fluorescence was noted in the other organs, such as heart, lung, liver, spleen, or kidney. **(C)**. The excised brain tissue administered with normoxia OM-MSCs and hypoxia OM-MSCs at 14 day after injection.

## Data Availability

All data has been included in the paper and supplement. The datasets used in this study are available from the corresponding author on reasonable request.

## Abbreviations

HE: Hematoxylin and eosin
ICH: Intracerebral hemorrhage
MSCs: Mesenchymal stem cells
miR-326: MicroRNA-326
miRNAs: MicroRNAs
mNSS: Modified neurologic severity scores
OM-MSCs: Olfactory mucosa MSCs
PTBP1: Polypyrimidine tract-binding protein 1
3-MA: 3-Methyladenine
