# Supplementary material for: Hypoxic preconditioning rejuvenates mesenchymal stem cells and enhances neuroprotection following intracerebral hemorrhage via the miR-326-mediated autophagy
Source: Stem Cell Res Ther. 2021 Jul 22;12:413. doi: 10.1186/s13287-021-02480-w (PMC8296710; doi:10.1186/s13287-021-02480-w)
Supplement: Supplementary file 1 — Additional file 1: Table S1. Groups used for the in-vitro experiments. [file 13287_2021_2480_MOESM1_ESM.docx]

**Table S1. Groups used for the in-vitro experiments.**

| **Groups** | **Step 1** | **Time (h)** | **Step 2** | **Time (h)** | **Step 3** | **Time (h)** | **Step 4** | **Time (h)** |
| --- | --- | --- | --- | --- | --- | --- | --- | --- |
| Normoxia | Untreated | 0 | Untreated | 0 | Untreated | 0 | Hemin | 18 |
| Hypoxia | Untreated | 0 | Untreated | 0 | Hypoxia | 48 | Hemin | 18 |
|  |  |  |  |  |  |  |  |  |
| Normoxia + mimics NC | Transfected with mimics NC | 24 | Untreated | 0 | Untreated | 0 | Hemin | 18 |
| Normoxia + miR-326 mimics | Transfected with miR-326 mimics | 24 | Untreated | 0 | Untreated | 0 | Hemin | 18 |
| Hypoxia + inhibitor NC | Transfected with inhibitor NC | 24 | Untreated | 0 | Hypoxia | 48 | Hemin | 18 |
| Hypoxia + miR-326 inhibitor | Transfected with miR-326 inhibitor | 24 | Untreated | 0 | Hypoxia | 48 | Hemin | 18 |
|  |  |  |  |  |  |  |  |  |
| Normoxia + miR-326 mimics + 3-MA | Transfected with miR-326 mimics | 24 | 3-MA treatment | 6 | Untreated | 0 | Hemin | 18 |
| Hypoxia + miR-326 inhibitor + rapamycin | Transfected with miR-326 inhibitor | 24 | rapamycin treatment | 6 | Hypoxia | 48 | Hemin | 18 |
|  |  |  |  |  |  |  |  |  |
| Normoxia + miR-326 mimics + control vector | Transfected with miR-326 mimics | 24 | Transfected with control vector | 24 | Untreated | 0 | Hemin | 18 |
| Normoxia + miR-326 mimics + PTBP1 vector | Transfected with miR-326 mimics | 24 | Transfected with PTBP1 vector | 24 | Untreated | 0 | Hemin | 18 |
| Normoxia + miR-326 mimics + PI3K activator | Transfected with miR-326 mimics | 24 | 740 Y-P treatment | 24 | Untreated | 0 | Hemin | 18 |
|  |  |  |  |  |  |  |  |  |
