# Supplementary figures and images for: Hypoxic preconditioning rejuvenates mesenchymal stem cells and enhances neuroprotection following intracerebral hemorrhage via the miR-326-mediated autophagy
Source: Stem Cell Res Ther. 2021 Jul 22;12:413. doi: 10.1186/s13287-021-02480-w (PMC8296710; doi:10.1186/s13287-021-02480-w)

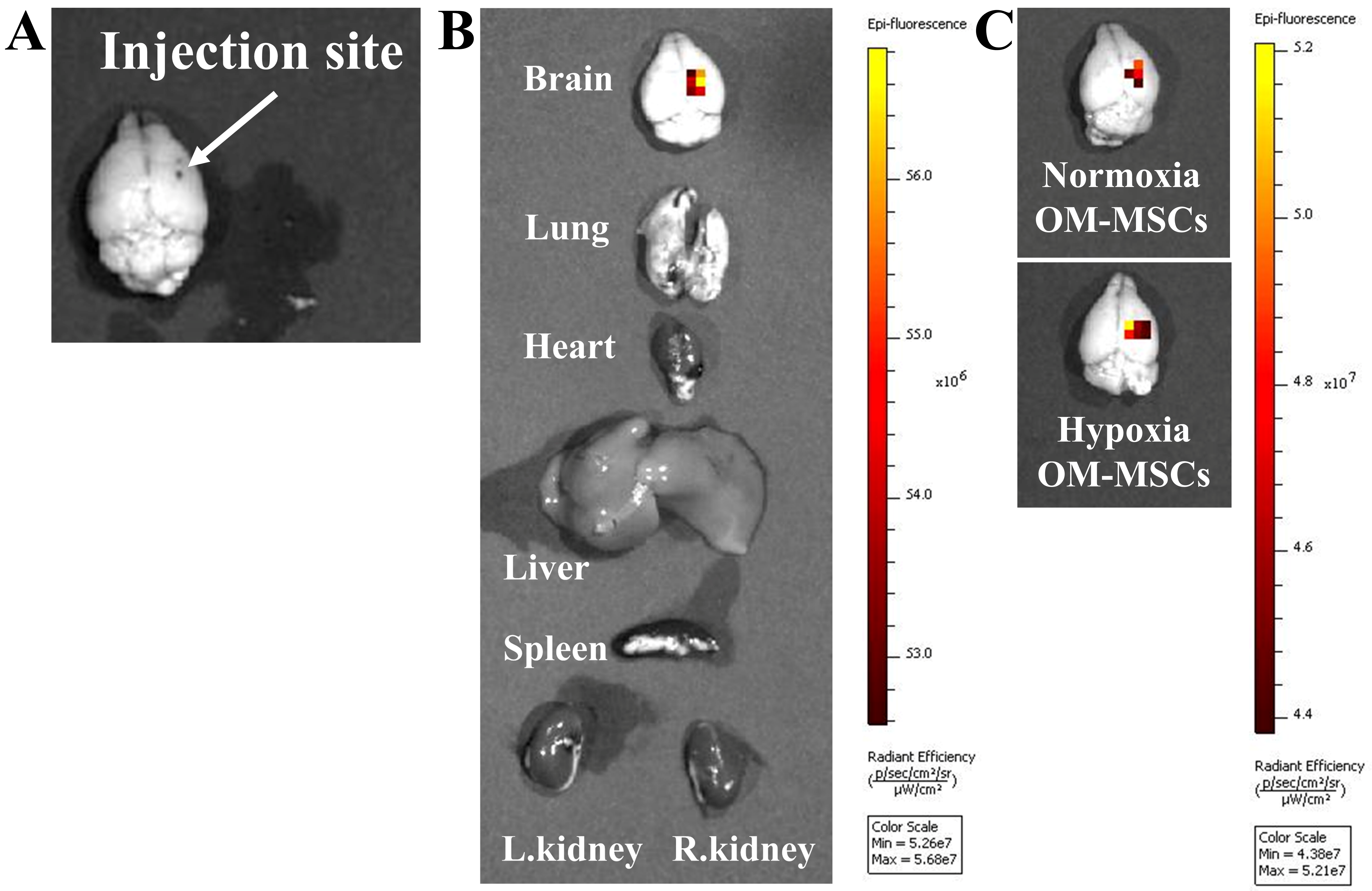

Supplement: Supplementary file 2 — Additional file 2: Figure S1. Detection of OM-MSCs with an in vivo imaging system (IVIS). (A). The injection site of collagenase IV and OM-MSCs. (B). In the excised organs at 14 day after injection, strong fluorescent signals were observed in the brain tissues. No fluorescence was noted in the other organs, such as heart, lung, liver, spleen, or kidney. (C). The excised brain tissue administered with normoxia OM-MSCs and hypoxia OM-MSCs at 14 day after injection. [file 13287_2021_2480_MOESM2_ESM.tif]
